# Pan-cancer analysis of *ARID1A* Alterations as Biomarkers for Immunotherapy Outcomes

**DOI:** 10.7150/jca.41296

**Published:** 2020-01-01

**Authors:** Tao Jiang, Xiaoxia Chen, Chunxia Su, Shengxiang Ren, Caicun Zhou

**Affiliations:** 1 Department of Medical Oncology, Shanghai Pulmonary Hospital & Thoracic Cancer Institute, Tongji University School of Medicine, Shanghai, China.; 2 Department of Pulmonary Medicine, Shanghai Respiratory Research Institute, Zhongshan Hospital, Fudan University, Shanghai, China.; 3 These authors contributed equally to this paper.

**Keywords:** *ARID1A*, Immunotherapy, Biomarker, Pan-cancer.

## Abstract

*ARID1A* alterations would compromise mismatch repair pathway and increase the number of tumor-infiltrating lymphocytes and PD-L1 expression in some cancers, which would cooperate with immune checkpoint inhibitors (ICIs) treatment. However, a comprehensive analysis of *ARID1A* alteration frequency and its predictive value for ICI treatment outcome in cancers has not yet been investigated. Hence, we performed this pan-cancer analysis to evaluate the prevalence and predictive value of *ARID1A* alterations across >40,000 cases in multiple cancer types. We found a high frequency (6.2%) of *ARID1A*, which were associated with significantly higher tumor mutation burden level across various cancers. Importantly, patients with *ARID1A* alterations and advanced cancers had the substantially prolonged overall survival in ICI treatment cohort, suggesting it might be used to predict a survival benefit from ICI therapy across multiple cancer types. Notably, *ARID1A* alterations were correlated with markedly high immune infiltrates in endometrial, stomach and colon cancer. However, patients with *ARID1A*-mutant renal clear cell carcinoma had dramatically lower CD8^+^ T cell infiltrations than those without, indicating the association between *ARID1A* alterations and immune infiltrates was cancer-dependent. Collectively, our findings highlight the important value of* ARID1A* alterations as pan-cancer predictive biomarkers for ICI treatment.

## Introduction

Although immune checkpoint inhibitors (ICIs) targeting programmed cell death protein 1 (PD-1), its ligand (PD-L1), or cytotoxic T lymphocyte antigen-4 (CTLA-4) significantly extend overall survival (OS) in patients with diverse types of cancer, it could benefit only a limited subpopulation of patients^1^. Hence, there is an urgent need to investigate the rational and effective strategies to improve the efficacy of ICIs in patients with cancer. Understanding genomic correlates of response to ICIs could help the development of novel biomarkers and therapies to enhance the clinical response and expand the benefit population^2^, ^3^.

The AT-rich interaction domain 1A (*ARID1A*) is a subunit of the chromatin remodeling complex SWI/SNF, which facilitates access of proteins to DNA. *ARID1A* is one of the most commonly mutated genes in cancer^4^, ^5^. A recent study found that *ARID1A* loss and impaired *ARID1A* binding to the mismatch repair (MMR) protein MSH2 similarly reduced MMR and increased mutation frequency and the number of tumor-infiltrating lymphocytes and PD-L1 expression. In mice with *ARID1A*-deficient ovarian cancer, PD-L1 inhibitor resulted in reduced tumor volume and better survival compared with controls^6^. Then, several retrospective and exploratory studies reported the relationship between *ARID1A* alterations and clinical benefit to ICI in some cancers. These findings suggested that *ARID1A* alteration would not only cooperate with ICI treatment but also have the potential predictive value for ICI therapy. However, a comprehensive analysis of *ARID1A* alteration frequency and its predictive value for ICI treatment outcome in diverse cancers has not yet been investigated.

In this study, we performed this pan-cancer analysis by using online database to systematically characterize the prevalence and predictive value of *ARID1A* alterations across multiple cancer types. We found a relatively high frequency (6.2%) of *ARID1A* alterations and significant predictive value for ICIs treatment in >40,000 patients with cancers. We also investigated the association between *ARID1A* alterations and tumor mutation burden (TMB) level or immune cell infiltrations. The current evidence suggested that *ARID1A* alterations would yield promising predictive value for ICI treatment across diverse cancers.

## Results and discussion

### Genetic alterations of *ARID1A* and its association with TMB level

**Figure 1:**
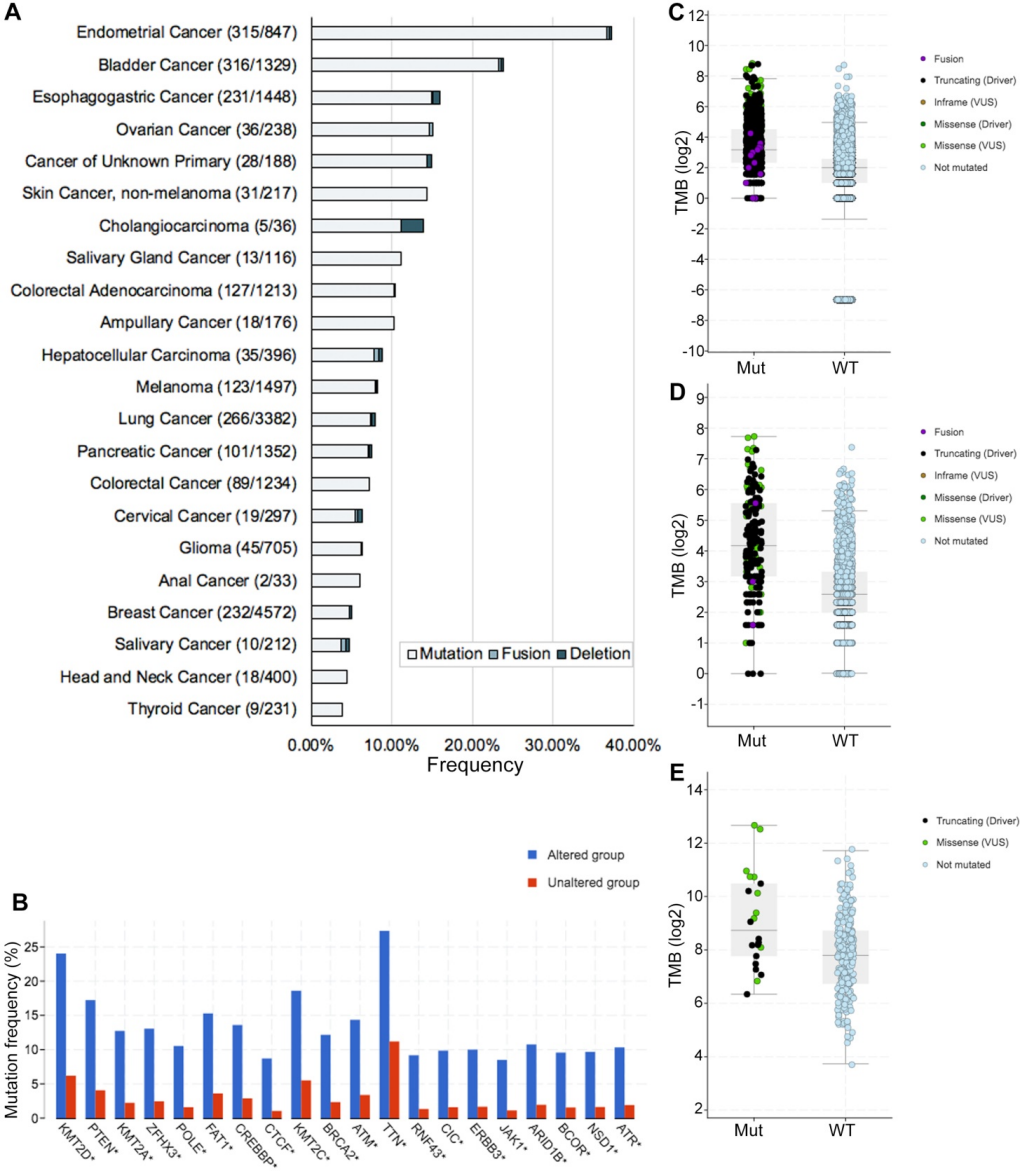
** Genetic alterations of *ARID1A* and its association with TMB level. A.** Prevalence of *ARID1A* alterations in different cancer types; **B.** Co-occurring of genetic mutations in cancers with *ARID1A* alterations; **C.** The association between TMB and *ARID1A* mutations in MSK-IMPACT cohort; **D.** The association between TMB and *ARID1A* alterations in immune checkpoint inhibitors treatment cohort; **E.** The association between TMB and *ARID1A* alterations in patients with microsatellite-stable solid tumors received immune checkpoint inhibitors treatment. TMB, tumor mutation burden; Mut, mutation; WT, wild type.

In this study, we defined *ARID1A* alterations as all kinds of nonsynonymous mutations including missense, frame-shift, splice site, nonstop, nonsense, fusions, deletions and translation start site changes. The frequency of *ARID1A* alterations in 40167 patients with various cancers was 6.2% (Figure 1A), with patients with endometrial cancer having the highest levels of *ARID1A* alterations (37.2%). Most of the alterations were missense mutations (39.6%, 979/2471; Supplemental Figure S1). The prevalence and spectrum of *ARID1A* alterations were slightly different in early-stage (TCGA cohort; Supplemental Figure S1A) versus advanced-stage cancers (MSK-IMPACT cohort; Supplemental Figure S1B). Co-occurring of genetic mutations in cancers with *ARID1A* alterations were not uncommon and some of them are popular driver genes in cancers (e.g. *KMT2D/A/C, PTEN, POLE*, etc.) (Figure 1B). Of note, co-occurring landscape of genetic mutations in cancers with *ARID1A* alterations was totally distinct in early-stage (TCGA cohort; Supplemental Figure S2A) versus advanced-stage cancers (MSK-IMPACT cohort; Supplemental Figure S2B). Considering *ARID1A* alterations promoting cancer mutability^6^, ^7^, we then investigate the difference of TMB level between *ARID1A* alteration and wild type groups. We selected a subset generated from MSK-IMPACT cohort that ensure the TMB could be comparable^8^. In the MSK-IMPACT cohort^8^, 10945 samples were identified and 912 (8.3%) of them had* ARID1A* alterations. TMB of patients with *ARID1A* alterations was significantly higher than it in those without (9 vs. 4 mutations/Mb,* P* < 0.0001; Figure 1C). This was validated in two ICI-treated cohorts (*P* < 0.0001, *P* = 0.0012, respectively; Figure 1C and D)^3^, ^9^. Notably, cancers with multiple *ARID1A* alterations had the highest TMB level (Supplemental Figure S2C-E). These results were consistent with a recent study, which also reported that the mutation load was significantly elevated in *ARID1A*-mutant tumors^6^. Together, these findings reveal a high prevalence of *ARID1A* alterations and its close relationship with TMB level across cancer types, suggesting that *ARID1A* alterations could be considered as biomarkers when conducting ICI treatment.

### Predictive and prognostic value of *ARID1A* alterations

**Figure 2:**
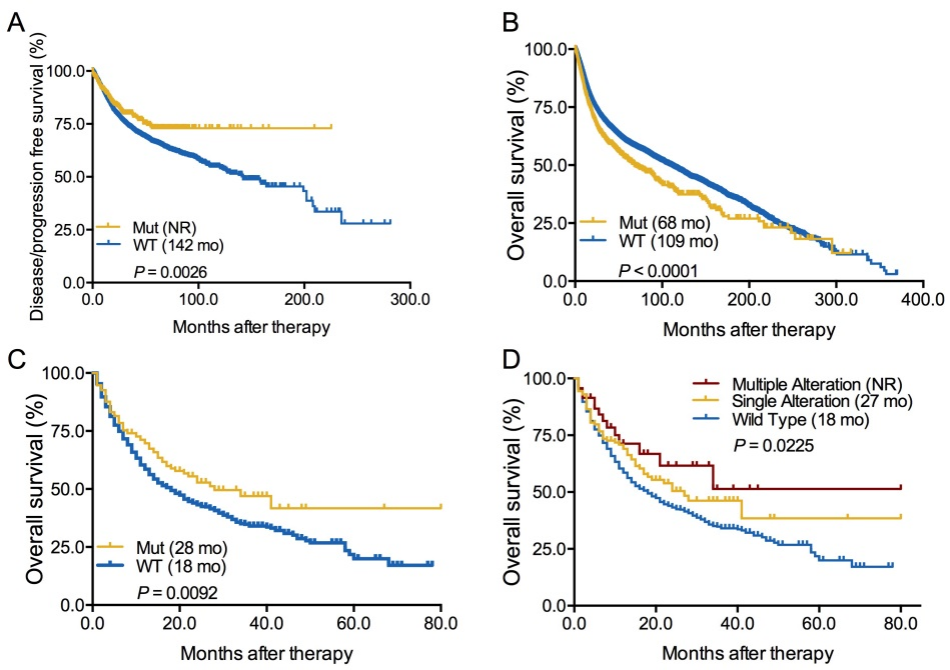
** Predictive and prognostic value of *ARID1A* alterations. A.** Predictive value of *ARID1A* alterations in all cancers;** B.** Prognostic value of *ARID1A* alterations in all cancers; **C.** Predictive value of *ARID1A* alterations in patients received ICI therapy;** D.** Subgroup analysis the predictive value of *ARID1A* alterations subtypes in patients received ICI treatment. Mut, mutation; WT, wild type.

Next, we evaluated the association between *ARID1A* alterations and clinical outcome in both whole group and ICI-treated cohort. We firstly found that patients with *ARID1A* alterations showed a significantly longer disease-free survival or progression-free survival [DFS/PFS, not reached vs 142 months, hazard ratio (HR) = 0.74, 95% confidence interval (CI) 0.64-0.91, *P* = 0.0026; Figure 2A] but markedly shorter OS (68 vs 109 months, HR = 1.30, 95% CI 1.22-1.47, *P* < 0.0001; Figure 2B) than those without in whole group. In early-stage cancers, *ARID1A* alterations were also correlated with longer DFS/PFS (*P* = 0.0005; Supplemental Figure S3A). However, the prognostic value of *ARID1A* alterations was only found in advanced-stage cancers (*P* = 0.0094; Supplemental Figure S3C) instead of early-stage cancers (*P* = 0.2086; Supplemental Figure S3B). In the ICI treatment cohort^9^, we firstly identified 1661 patients with different cancers receiving ICI therapy and 193 of them with *ARID1A* alterations (Supplemental Table S1). Interestingly, patients with *ARID1A* alterations had a substantially prolonged OS of 28 months vs 18 months in the wild-type group (HR = 0.73, 95% CI 0.61-0.93, *P* = 0.0092; Figure 2C). Subgroup analysis revealed that patients with multiple *ARID1A* alterations had the longest OS than those with single *ARID1A* alterations or without (not reached vs. 27 vs. 18 months, *P* = 0.0225; Figure 2D). Of note, we did not observe the association between* ARID1A* alterations and OS in patients with microsatellite-stable (MSS) solid tumors (*P* = 0.4075; Supplemental Figure S4A). Even in patients with multiple *ARID1A* alterations and MSS solid tumors, we also did not found the close association (*P* = 0.4899; Supplemental Figure S4B). These findings were consistent with previous publications that *ARID1A* alterations contributes to impaired MMR and mutator phenotype in cancer, and may cooperate with ICI treatment^6^.

### Immune landscape of cancer with *ARID1A* alterations

**Figure 3:**
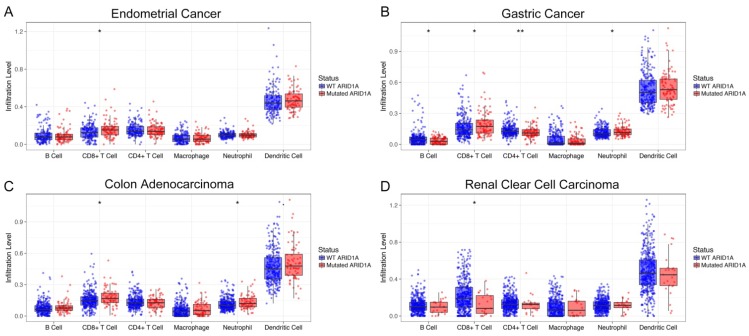
** Immune landscape of cancer with *ARID1A* alterations.** The association between *ARID1A* alterations and six immune infiltrates in** A.** Endometrial cancer;** B.** Stomach carcinoma; **C.** Colon adenocarcinoma; **D.** Renal clear cell carcinoma.

To unravel the potential mechanism of the predictive value of *ARID1A* alterations for ICI treatment, we then surveyed the relationship between *ARID1A* alterations and six common immune infiltrates (B cells, CD4^+^ T cells, CD8^+^ T cells, macrophages, neutrophils and dendritic cells) across different cancer types^10^. Interestingly, *ARID1A* deficiency was correlated with significantly higher six immune infiltrates in most of the cancer types, especially CD8^+^ T cells, including endometrial cancer (Figure 3A), stomach carcinoma (Figure 3B) and colon adenocarcinoma (Figure 3C). However, patients with *ARID1A*-mutant renal clear cell carcinoma had dramatically lower CD8^+^ T cell infiltrations than those without (Figure 3D). Of note, copy number variations (either deletion or amplification) of *ARID1A* were associated with substantially lower six immune infiltrates in most of the cancer types including endometrial cancer, gastric cancer, colon adenocarcinoma, head and neck cancer, lung squamous cell carcinoma and breast invasive carcinoma (Supplemental Figure S5).

## Conclusions

To our knowledge, this study firstly reported a high frequency of *ARID1A* alterations and the predictive significance of *ARID1A* alterations for ICI treatment in multiple cancer types. We also observed that *ARID1A* alterations were associated with significantly higher TMB level. Although *ARID1A* alterations were correlated with significantly inferior OS in total populations, they were associated with significantly prolonged OS in ICI treatment cohort, suggesting it might be used to predict a survival benefit from ICI therapy across multiple cancer types. Notably, patients with* ARID1A* alterations were correlated with markedly high immune infiltrates in endometrial, stomach and colon cancer but dramatically lower CD8^+^ T cell infiltrations in* ARID1A*-mutant renal clear cell carcinoma, indicating the association between *ARID1A* alterations and immune infiltrates was cancer-dependent. Collectively, our findings highlight the important value of* ARID1A* alterations as pan-cancer predictive biomarkers for ICI treatment.

## Supplementary Material

Supplementary figures and tables.

## Abbreviations

*ARID1A*: AT-rich interaction domain 1A
CI: confidence interval
CTLA-4: cytotoxic T lymphocyte antigen-4
HR: hazard ratio
ICIs: immune checkpoint inhibitors
MMR: mismatch repair
MSS: microsatellite-stable
OS: overall survival
PD-1: programmed cell death protein 1
PD-L1: PD-1 ligand
TMB: tumor mutation burden
