# Supplementary material for: Pan-cancer analysis of ARID1A Alterations as Biomarkers for Immunotherapy Outcomes
Source: J Cancer. 2020 Jan 1;11(4):776–80. doi: 10.7150/jca.41296 (PMC6959029; doi:10.7150/jca.41296)
Supplement: Supplementary file 1 — Supplementary figures and tables. [file jcav11p0776s1.pdf]

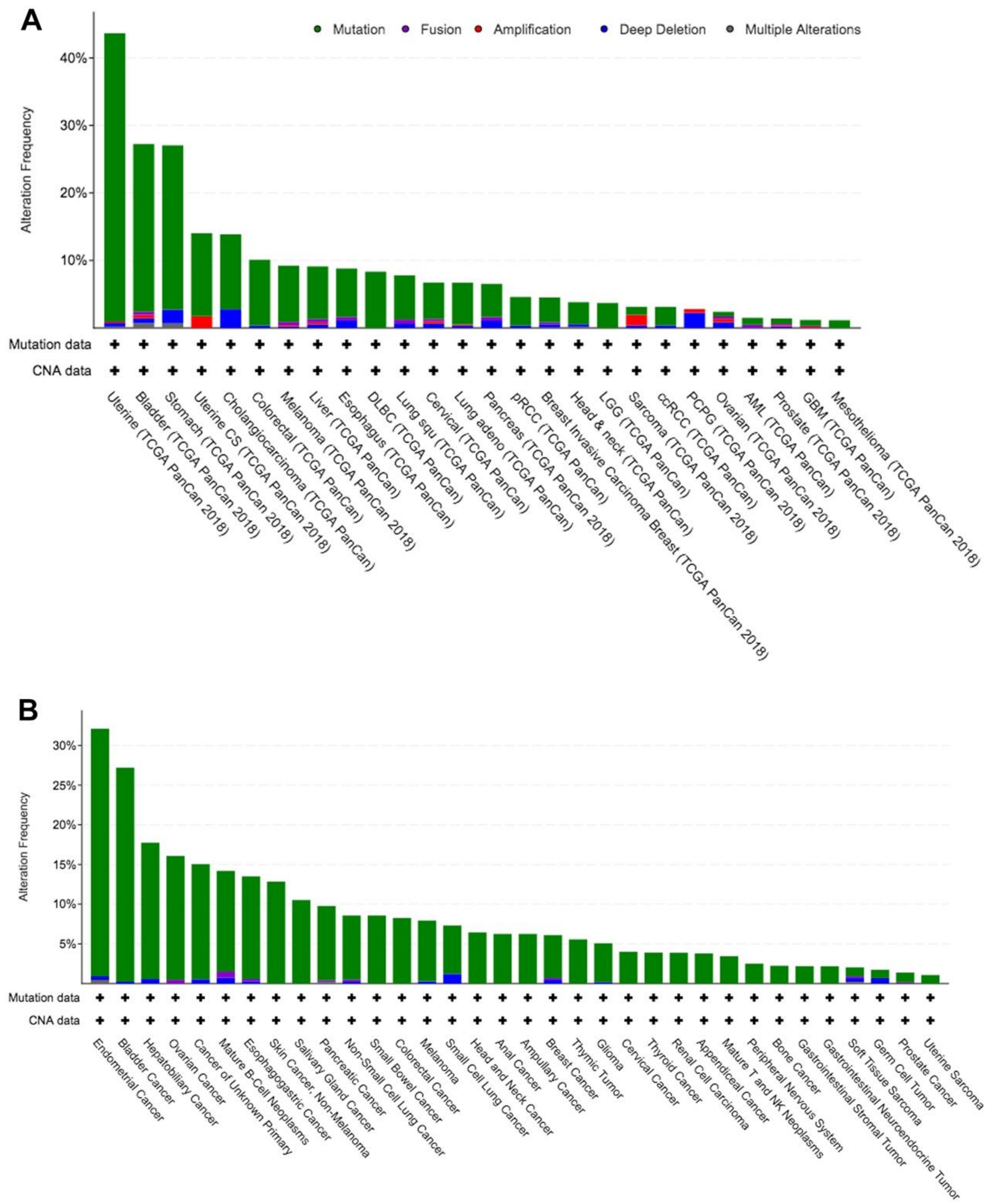

Figure S1. The frequency of ARID1A alterations in early-stage cancer (A) and advanced-stage cancer (B).

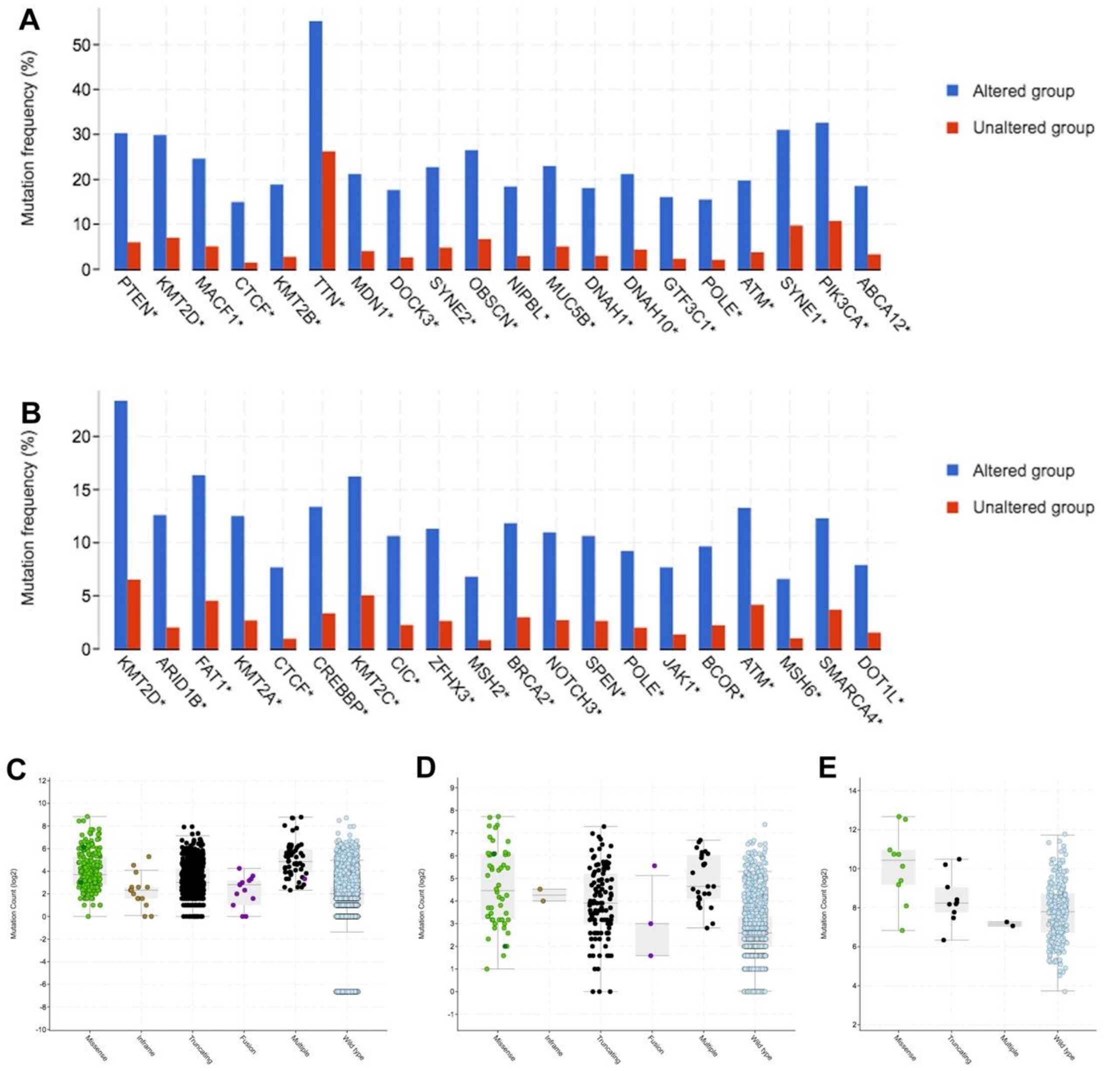

Figure S2. Co-occurring of genetic mutations in early-stage cancer (A) and advanced-stage cancer (B) with *ARID1A* alterations; The association between TMB and *ARID1A* alterations subtypes in MSK-IMPACT cohort (C), 1661 patients with advanced cancer received immune checkpoint inhibitors treatment cohort (D), 249 patients with microsatellite-stable solid tumors received immune checkpoint inhibitors treatment (E).

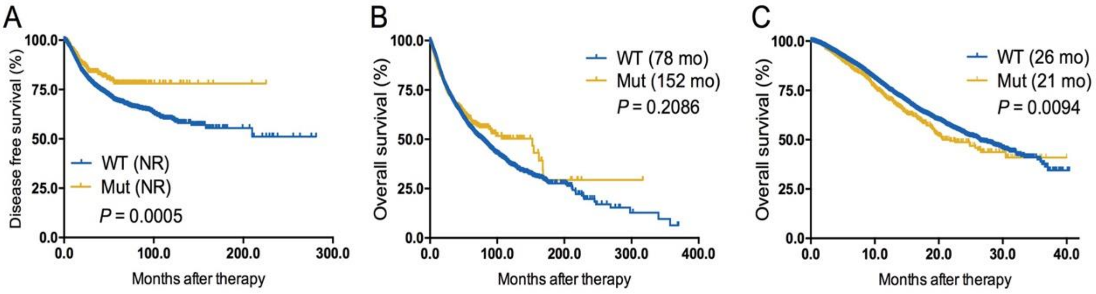

Figure S3. Predictive value of *ARID1A* alterations in TCGA cohort (A); Prognostic value of *ARID1A* alterations in TCGA cohort (B); Prognostic value of *ARID1A* alterations in MSK-IMPACT cohort (C).

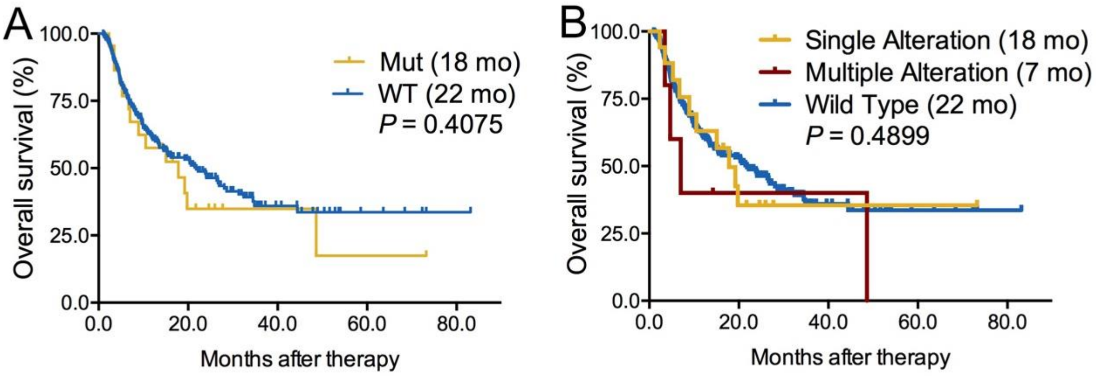

Figure S4. A. Predictive value of *ARID1A* alterations in patients with microsatellite-stable solid tumors received ICI therapy; B. Subgroup analysis the predictive value of *ARID1A* alterations subtypes in patients microsatellite-stable solid tumors received ICI treatment.

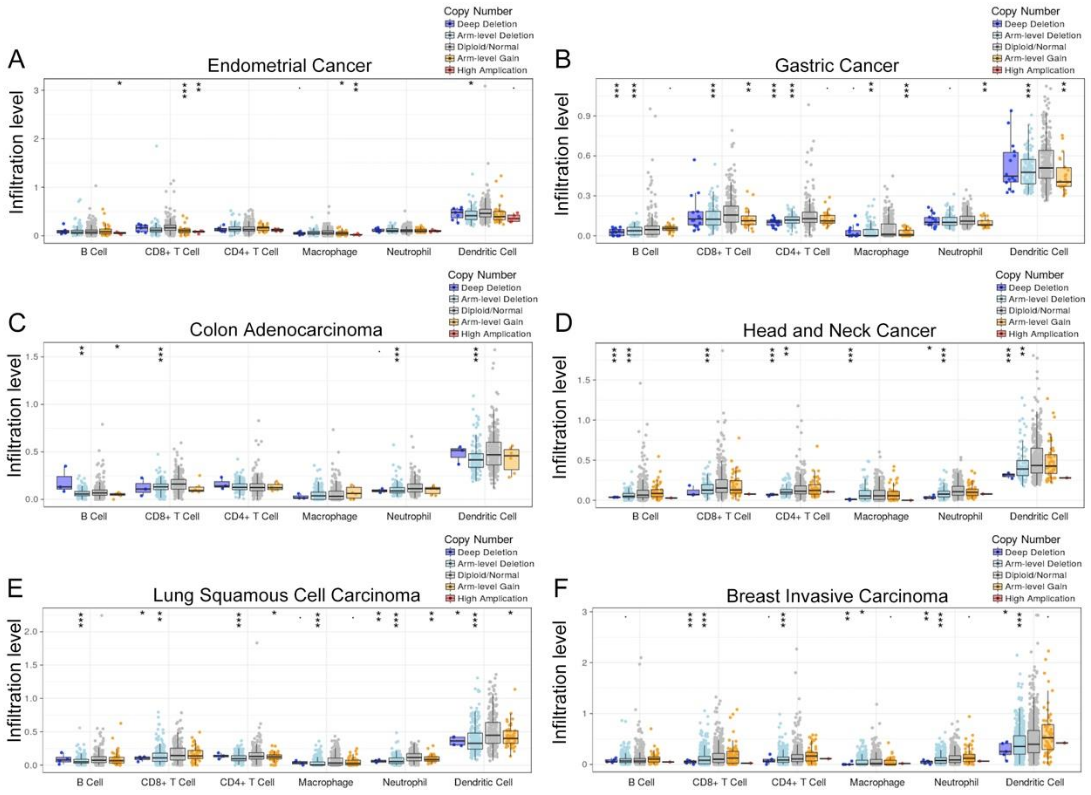

Figure S5. The association between *ARID1A* copy number variations and immune infiltrates in endometrial cancer (A), gastric cancer (B), colon adenocarcinoma (C), head and neck cancer (D), lung squamous cell carcinoma (E) and breast invasive carcinoma (F).
